# The collαgen III fibril has a “flexi-rod” structure of flexible sequences interspersed with rigid bioactive domains including two with hemostatic roles

**DOI:** 10.1371/journal.pone.0175582

**Published:** 2017-07-13

**Authors:** J. Des Parkin, James D. San Antonio, Anton V. Persikov, Hayat Dagher, Raymond Dalgleish, Shane T. Jensen, Xavier Jeunemaitre, Judy Savige

**Affiliations:** 1 From the University of Melbourne Department of Medicine (Northern Health), Melbourne, VIC, Australia; 2 Operations, Stryker Global Quality and Operations, Malvern, PA, United States of America; 3 Lewis-Sigler Institute for Integrative Genomics, Princeton University, Carl Icahn Lab, Princeton, NJ, United States of America; 4 Department of Genetics, University of Leicester, Leicester, United Kingdom; 5 Wharton Business School, University of Pennsylvania, Philadelphia, PA, United States of America; 6 INSERM U970 Paris Cardiovascular Research Centre, Paris France; 7 University Paris Descartes, Paris Sorbonne Cite, Paris, France; University of Akron, UNITED STATES

## Abstract

Collagen III is critical to the integrity of blood vessels and distensible organs, and in hemostasis. Examination of the human collagen III interactome reveals a nearly identical structural arrangement and charge distribution pattern as for collagen I, with cell interaction domains, fibrillogenesis and enzyme cleavage domains, several major ligand-binding regions, and intermolecular crosslink sites at the same sites. These similarities allow heterotypic fibril formation with, and substitution by, collagen I in embryonic development and wound healing. The collagen III fibril assumes a “flexi-rod” structure with flexible zones interspersed with rod-like domains, which is consistent with the molecule’s prominence in young, pliable tissues and distensible organs. Collagen III has two major hemostasis domains, with binding motifs for von Willebrand factor, α2β1 integrin, platelet binding octapeptide and glycoprotein VI, consistent with the bleeding tendency observed with *COL3A1* disease-causing sequence variants.

## Introduction

The collagens are the major proteins of the extracellular matrix. Each molecule is a homo- or heterotrimer of three α chains with G-X-Y repeats. Twenty-eight different collagens have been identified with 46 distinct chains [1], that serve as scaffolds for the attachment of cells and matrix proteins, but are also biologically active.

**Fig 1:**
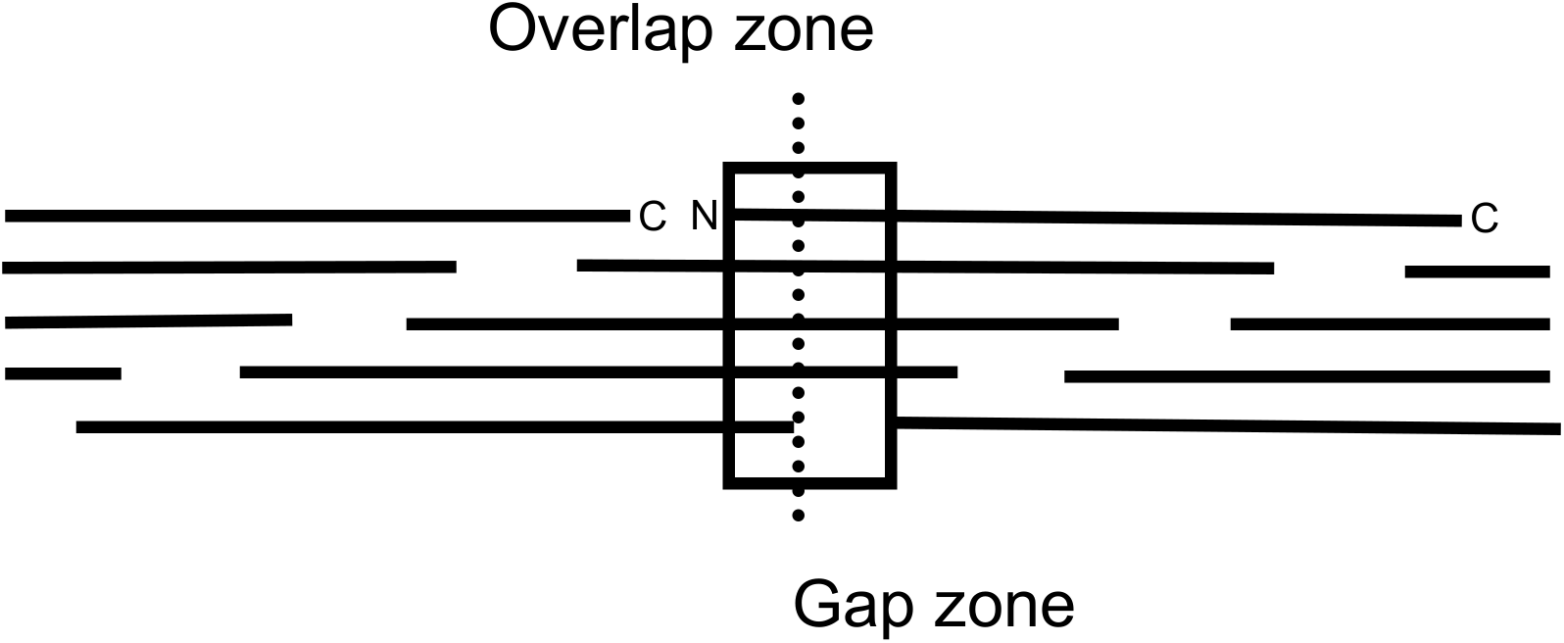
Arrangement of five overlapping collagen III α1 chains in the fibril D-period with overlap and gap zone indicated.

Collagen I is the most abundant collagen, and a heterotrimer of two α1(I) and one α2(I) chains [2]. Its fibrils have a characteristic banding pattern on heavy metal staining because of their charged residues [3, 4]. The basic repeating structure of the fibril is the D-period, 67 nm long, composed of one region of complete molecular overlap (overlap zone) and one of incomplete overlap (gap zone). Each D-period contains the complete collagen monomer (M) sequence derived from overlapping consecutive elements designated as M 1–5, where M1 is the most N-terminal and M5 the most C-terminal segment of the collagen sequence (Fig 1).

Collagen III is another fibrillar collagen. It can form heterofibrils with collagen type I, and is often replaced by collagen I in embryonic development and wound healing [5]. It is a homotrimer of three identical α1(III) chains, that together with the collagen I α1(I) and α2(I) chains are members of the A clade [6], and whose corresponding genes (*COL3A1*, *COL1A1* and *COL1A2)* have arisen from a common progenitor.

The three collagen III α1 chains are cross-linked in a staggered array through lysine residues at four sites on each molecule to form microfibrils [7]. These fibrils demonstrate the same periodic banding as seen with collagen I. Fibril size varies with tissue type and developmental stage, but overall, the fibrils are finer than for collagen I and recognized on staining as reticulin [8].

Collagen III co-localizes with collagen I in many tissues including the vasculature, bowel and skin [9]. It is generally less abundant than collagen I, except in the walls of distensible organs and blood vessels [10], but its fibril cross-links and proteoglycan-rich matrix contribute mechanical strength and distensibility [11]. Collagen III also participates in a variety of biological functions. It has critical roles in cell-binding, hemostasis and angiogenesis [12], tissue remodeling, fetal development [13], and wound healing [14, 15]. It is affected by microbial infections, ageing, diabetes, and inherited disease. Disease-causing *COL3A1* variants result predominantly in vascular Ehlers-Danlos syndrome (EDS type IV) (MIM 130050). This affects one in 150,000 individuals, and is autosomal-dominantly inherited [16]. Clinical features include easy bruising, and arterial, intestinal or uterine rupture (15). The atypical form, acrogeria, is characterized by translucent skin and premature aging of the face and peripheries [16,17], but these features are present to some extent in all affected individuals. *COL3A1* variants have also been associated with cerebral aneurysms [18], gastroesophageal reflux disease [19], and pelvic organ prolapse [20].

Interactomes are protein maps which indicate structural features and the sites of interactions with other molecules. The unique molecular structure of collagens, with their predominantly rigid, triple helical conformation, allows the construction of maps wherein triple helical domains can be represented as 2D linear arrays of three polypeptide chains. The collagen I interactome has been used to deduce functional and disease-associated domains from ligand-binding data [21,22,23]. Here we have constructed and analyzed the collagen III interactome. Note that databases exist which archive disease variants mapping to the collagens (http://www.le.ac.uk/genetics/collagen/), or through which researchers can access and plot data of interest onto maps of fibrillar collagens, including collagen III [24]. However, to date a comprehensive analysis of all known ligand binding sites and disease variants for collagen III has not been reported.

## Results

The amino acid numbering systems for procollagen III and the mature collagen III protein that are used here are shown in S1 Table.

### D-period and banding pattern

**Fig 2:**
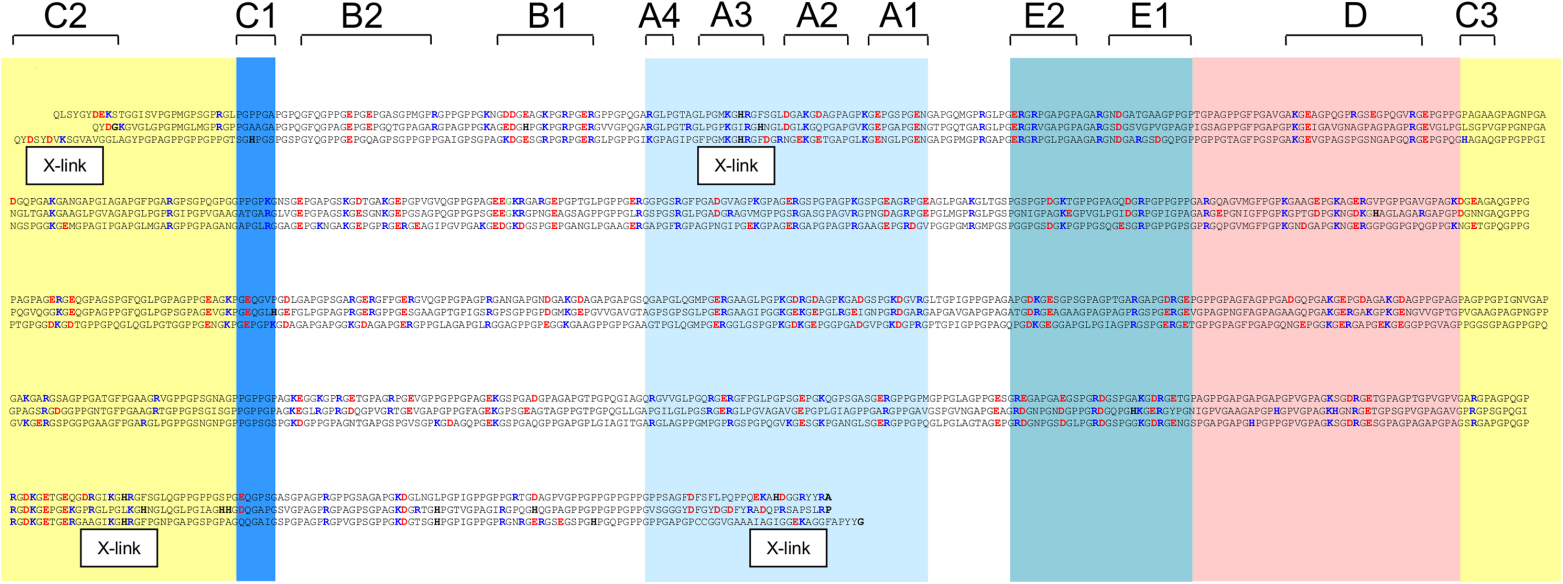
Arrangement of collagen I α1 (top), α2 (middle) and collagen III α1 (bottom) chains demonstrates approximate alignment of charge across all three molecules in the D-period.

When the cross-linking K residues in the collagen α1(III) chain were aligned with those in the collagen α1(I) and α2(I) chains, collagen III had a nearly identical D-period arrangement to collagen I (Figs 1 and 2), with the major crosslink pairs being separated by 843 residues in both collagen types [7,25].

When the D-periods of the α1(III) chain were examined for positive- (R, K) and negatively- charged (E, D) residues, the charge, although not necessarily the residue type, was typically in register across the D-period, and also with the α1(I) and α2(I) chains, and hence with the collagen I D-period (Fig 2). The charge was not however symmetrical about the molecules’ midpoints which implies unidirectional alignment of collagen III and I during their assembly into heterotypic fibrils. Charge alignment meant that binding motifs for some ligands, such as proteoglycans and HSP47, are also in the same locations for both collagens.

### Analysis of fibril stability and structure

**Fig 3:**
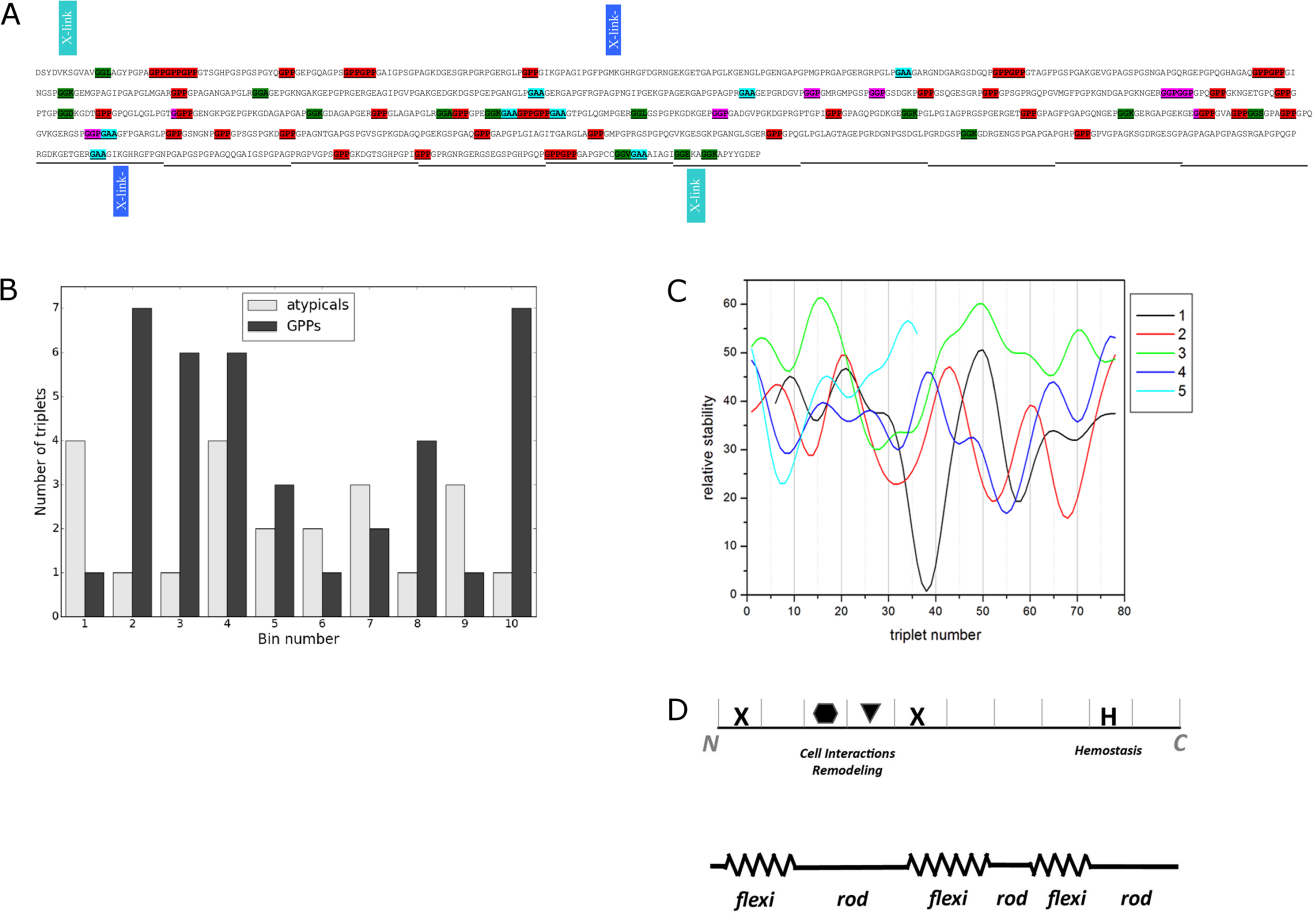
a. Collagen III D-period demonstrating location of GPP residues (in red) proposed to confer stability and atypical triplets (GAA, GGP, etc, in other colors) proposed to contribute flexibility.; b. Each monomer of the collagen III D-period was divided into ten bins of equal size (about 23 residues) starting at the N-terminus. The number of GPP triplets and atypical triplets (GAA, GGP, etc) were added for all five monomers and plotted against bin number; c. Relative triple helix stability for amino acid sequences of each monomer within the collagen III D-period was predicted using the collagen stability calculator (http://semimajor.net/collagen_calculator/). Monomers 1–5 represent five segments of the collagen III sequence, beginning at the N-terminus. Three comprise approximately 234 amino acid residues but the two that included the gap zone were shorter. Examining these stability curves together suggests that some regions of the D-period are more stable, and others less so, suggesting a “flexi-rod” model for collagen III; d.Top: Schematic of type III collagen monomer with sites for cell interactions and remodeling flanked by crosslinks (X) and hemostasis domain (H). Bottom: Stability modeling indicates clusters of atypical collagen sequences of lower stability (springs) are interspersed with rigid zones (rods) hosting sequences that carry out crucial biologic functions.

Collagen III has a higher G, and lower P content than the other fibrillar collagens [26], and nearly twice the number of atypical triplets as type I collagen (Fig 3). GPP-rich regions promote triple-helical rigidity with more compact 7/2 symmetry, while non-P (‘atypical’) residues alter the triple helical twist [27]. Thermodynamic studies on collagen peptides show that the GPP triplets are the major contributors to triple-helix stability, and that atypical triplets are destabilizing [28,29]. Atypical and GPP triplets tended to predominate in different bins of the collagen III molecule (Fig 3). While atypical triplets occurred more often in bins 1, 6, 7, and 9, GPP triplets were more common in bins 2, 3, 4, 8, and 10. There was a strong inverse correlation between atypical and GPP triplets with a Pearson correlation coefficient of -0.52. This suggested the presence of alternating rigid and flexible regions within the D-period, along the length of the collagen III molecule.

The collagen stability calculator demonstrated co-localization of local stability variations (Fig 3), with three major regions of decreased stability, including triplets 5–15, 30–40, and 50–60. We thus propose collagen III functions as a “flexi-rod” in which a confluence of atypical triplets creates flexible domains, allowing focal expansion or of several discrete fibril regions (Fig 3). Yet, two of the three flexible domains incorporated hydroxyl (OH)-K substrates for intermolecular crosslinking between triple helices. Thus the degree of flexibility of the collagen III fibril afforded by the confluence of atypical amino acid triplets may be modulated by the fibril’s extent of crosslinking and may vary, from highly flexible in young organisms or early in wound healing, to less flexible with the more highly cross-linked protein with ageing or later in scar formation. One of the flexible zones lacked the potential for intermolecular crosslink formation and fell in the middle of the gap zone, a thinner, more pliable fibril region. This suggested that the flexi-rod structure of collagen III, regardless of crosslinking, still confers some flexibility to the polymer.

The rod-like domains containing the major cell- and ligand- binding sites were located between the relatively flexible zones on collagen III (Fig 3). This may allow the molecule to preserve the triple helical conformations of the regions critical for biologic function.

A stability analysis of the type I collagen fibril also revealed a “flexi-rod” structure (data not shown). However, the more abundant atypical triplets in collagen III are consistent with a more flexible polymer, and with collagen III’s predominance in pliable tissues such as the skin of young animals, wounds, and in distensible organs.

### Sites related to structure, assembly, turnover, modification, cleavage and ligand-binding

**Fig 4:**
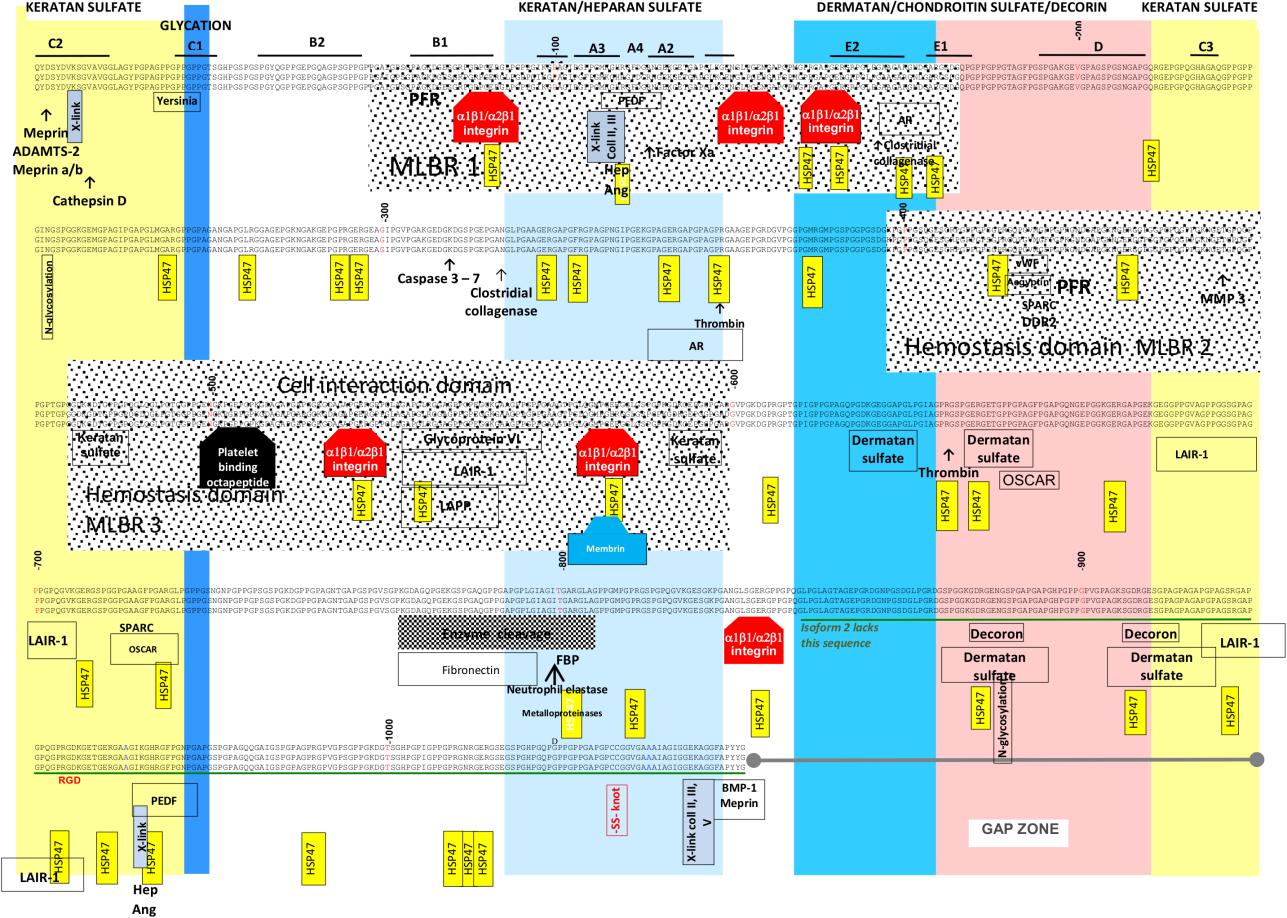
Human collagen III interactome demonstrating the positions of putative Cell interaction domain, Fibrillogenesis and enzyme cleavage domain, Major ligand-binding regions (MLBR1, 2 and 3), Hemostasis domains, and miscellaneous ligand binding sites and structural features on the collagen triple helix in the fibril’s D-period. Sequences or residues comprising binding sites or structural features are indicated by labels that appear beneath the relevant sequence or residue(s) and approximate their spans. Some enzyme cleavage sites are indicated by arrows pointed to the approximate center of the enzyme’s recognition sequence. Abbreviations used in the interactome for the first time include: ang, angiogenesis; AR, A-rich; coll, collagen; hep, heparin/heparan sulfates; FBP, fibronectin-binding protein; and PFR, platelet fibrinogen receptor.

These include N- and C-propeptides, A-rich sequences, F residues, C cross-links, K cross-links, O and N glycosylation sites, P glycosylation sites, and cleavage sites (N and C propeptidases, matrix metalloproteinases (MMP), serine proteases, chaperones, Secreted Protein Acidic and Rich in Cysteine (SPARC), and Discoidin domain receptors (DDR1 and DDR2) (Fig 4).

#### N- and C-propeptides

Collagen α1(III) has N- and C-propeptides that are normally cleaved. Retention of the quasi-rigid N-terminal propeptide potentially restricts fiber growth [5,7]. The collagen α1(III) C-propeptide includes the NC1 recognition sequence, GNPELPEDVLDVSSR [30], critical for triple helix nucleation [31], as well as a HtrA1 serine protease cleavage site [32].

#### A-rich sequences

Collagen III has two A-rich sequences, GAAGARGNDGAR and GPAGERGAPGPAGPRGAA that may contribute to elasticity [33]. Similar sequences occur at these locations in collagen I.

#### F residues

Collagen III has three major cross-fibril clusters of F residues in the same locations as in collagens II and III [34] where they are proposed to promote collagen monomer assembly into fibrils. For the collagen III sequence RGQPGVMGF, F is a crucial component of the DDR2, SPARC and vWF–collagen binding complexes [35,36,37].

#### C cross-links

The C residues at 1196–1197 in the collagen III α1 chains form a disulfide knot [38], that facilitates molecular realignment after denaturation. The mature collagen I protein does not have C residues which selects against trimerization with collagen III. Instead collagen I uses the C-terminal (GPP)_5_ motif.

#### K cross-links

Divalent and trivalent crosslinks covalently join collagens III and I [77]. The K covalent cross-links at residues 110 and 953 stabilize collagen III fibril formation. The other cross-link sites are in the collagen III N- and C-telopeptides [77].

#### O- and N-glycosylation sites

Collagen III has one O-glycosylation site [39], and N-glycosylation sites at the GXN motifs.

#### P hydroxylation sites

Most P residues in the Yaa position of the GXY triplet in collagen III are 4-hydroxylated. The GHPGPIGPPGPR motif that is 3-hydroxylated in collagen I and II is not hydroxylated in collagen III [77]. The absence of this cross-link may contribute to collagen III’s inability to form homotypic fibrils [77].

#### N and C propeptidases

Meprin a/b cleaves both the N and C-propeptides [40] of collagen III. The N-propeptide is also removed by ADAMTS-2, and the C-propeptide by procollagen C-proteinase, also known as bone morphogenetic protein 1 (BMP1) [40].

#### MMP

MMPs are important in extracellular matrix degradation and tissue remodeling, and activating other MMPs. Collagen III is cleaved by MMPs 1, 2, 3, 9 and 13 at residues 791–806, located about three quarters of the length of the molecule, near the fibrils gap zone [41,42]. There are also cleavage sites nearby for elastase, trypsin and thermolysin thought to act on the soluble, denatured protein [43].

#### Serine proteases and other enzymes

Collagen III α1 chains have consensus sequences for thrombin and factor Xa cleavage, which are potentially important for collagen III’s hemostatic role but may only be relevant to the denatured molecule [44].

#### Chaperones

HSP47 is a procollagen-specific chaperone that recognizes GXR where the R residue is critical [45]. HSP47 stabilizes the collagen triple helix, clamping the three chains in register. Each collagen III α1 chain binding site may have one or two chaperones bound to it, which prevents lateral aggregation of collagen molecules [45]. Not all of the predicted HSP47 binding sites may be functional.

#### SPARC

SPARC acts as a chaperone but also modulates collagen fibril assembly [46] and tissue remodeling. The minimal binding motif in several collagens is GVMGFO [36,46]. Collagen III and I have a common high affinity SPARC binding site but collagen I has an additional low affinity site [46].

#### Discoidin domain receptors (DDR1 and DDR2)

DDRs are tyrosine kinase receptors that induce the expression of MMPs and BMP, and potentially regulate collagen production and organization. Their main binding site in collagen III includes the GVMGFO motif that also binds SPARC, and contributes to the binding of vWF, overlaps with a conserved F residue, and is in the same location in collagen I [47].

### Isoform (P02461-2)

Expression of the collagen III isoform lacking C-terminal residues 694–996 (UniProt KB) has not been confirmed *in vivo*. It lacks the final monomer of the D-period, which includes the fibrillogenesis domain and sites for the disulfide knot, cross-links, and some ligands, but retains the K residues required for cross-linking.

### Binding sites for extracellular matrix molecules, integrins, cells and other ligands

These include the extracellular matrix molecules (proteoglycans, collagens, fibronectin, thrombospondin 4, integrins, RGD motif, leucocyte-associated immunoglobulin-like receptors (LAIR) 1 and 2, OSteoClast-associated Receptor (OSCAR) and other binding motifs.

#### Proteoglycans

The binding motifs for proteoglycans in collagen III are GE/DR/KGE/DXGXXGX [48] (Table 1). Binding sites for decoron (the core protein of decorin) and dermatan sulfate proteoglycan overlap with the motif KXGDRGE at the e1-d band junction [49]. These are at the same locations as in collagen I [22].

**Table 1:** Major structural features and ligand binding sites for collagen III.

Name	Motif	Sequence	Residues	Comments	Detection method	Reference
Structural features						
C cross-links	CC	CC	1043–1044	Forms bonds between collagen III α1 chains	X Ray crystallography	[38]
K cross-links	GMXGHR (bovine) IAGIGGEXA	GMKGHRGIKGHR IAGIGGEKA	108–113951–956 1052–60	Cross links collagen III and collagen I or II	Enzyme digestion and sequencing	[7,99]
Modification sites and sites related to assembly, trafficking and turnover						
4 P-hydroxylation	G-Xaa-P	N/A	Multiple sites	Stabilizes collagen triple helix	Protein sequence	[100]
O-linked glycosylation	PGMKGHRG	PGMKGHRG	107–114	Regulates cross-link maturation	Cyanogen bromide fragments, protein sequence	[39, 101]
N-linked glycosylation	NXaaS/T	NGS NGS	236–238 884–886	Unlikely in native collagen	Protein sequence	NetNGly 1.0
NC1 recognition sequence	GNPELPEDVLDVSSR	C-terminal propeptide	C-terminal to onset of triple helix	Highly conserved sequence, critical to triple helix nucleation	Protein sequence	[31]
HSP47	GXR	GXR	Multiple sites, in the same locations as collagen I α1 and α2	Procollagen-specific chaperone that facilitates folding in the ER	Protein sequence	[102]
Secreted Protein Acidic and Rich in C (SPARC)	GVMGFO GAAGFO	GVMGFO GAAGFP	423–428 717–722	Matricellular protein with roles in cell differentiation and wound healing	Rotary shadowing, cyanogen bromide, synthetic peptides	[46]
Membrin	MxxE	MPGE	574–577	Transports proteins within the Golgi	Protein sequence	Minimotif
Glycation sites	Select K residues	K	Undefined	Advanced glycation end-products may form at select residues	Sites in collagen I α1 (434) and α2 chains (453, 479, 924); cross-fibril glycation zone for type I/III fibrils, blue stripe, Fig 4.	[88]
Cleavage sites						
Procollagen III N-proteinase		NYSP/QYDS	.…/1-4	Cleaves native collagen III	Protein sequence	[103,104]
ADAMTS-2		NYSP/QYDS	…./1-4	N-propeptidase that cleaves collagens I, II III, probably distinct from procollagen III N-proteinase	Protein sequence	[104]
Meprin a/b	G/DEPMDF	NYSP/QYDS PYYG/DEPM	…./1-4 1065-1068/…	Cleaves both N- and C- propeptides	SDS-PAGE	[40]
Bone morphogenetic protein 1 (BMP1; Procollagen C-proteinase, PCP)	G/DEPMDF	C-propeptide	1065-1068/…	Cleaves C-propeptide	SDS-PAGE	[40]
Metalloproteinase-1 (MMP1, 3 and 9)	GPLGIAGITGARGLA	GPLGIAGITGARGLA	792–803	Cleaves collagen III ¾ along the molecule; activates other MMPs	Agarose gels and sequencing	[105]
MMP3 (stromelysin 1)	KNGETGPQGP	KNGETGPQGP	457–466	Cleaves collagen III ¾ along the molecule; activates other MMPs	Agarose gels and sequencing	[105]
High temperature requirement A 1 (HtrA 1) serine protease	GPVCFL/C	C-propeptide	C-terminal to triple helix	Serine protease, binds to C-terminus	Solid phase binding assay	[32]
Clostridium collagenase	LGPA	LGPA	160–163 325–328	Cleavage	Protein sequence	[83,84,106]
Caspase 1	WTD/SS	WTD/SS in C- propeptide	C-terminal to triple helix	Cysteine-aspartic protease that activates proteins by cleavage, digests ECM	Protein sequence	[107]
Caspase 3–7	DGKDG	DGKDG	311–315	Cysteine-aspartic protease, involved in apoptosis and digests extracellular matrix	Protein sequence	[107]
Cathepsin D	KSGVAGGI (bovine)	KSGVAGGL	8–16	Non-specific tissue proteinase that	Protein sequence	[108]
Caspase 3–7	DGKDG	DGKDG	311–315	Cysteine-aspartic protease, involved in apoptosis an ddiegests ECM	Protein sequence	[107]
Neutrophil elastase	I/T	IT	799/800 and 800/801	Cleaves in MMP enzyme cleavage domain	Protein sequence	[69]
Trypsin	R/G	RG	Multiple sites	Cleaves in MMP enzyme cleavage domain	Protein sequence	[109]
Thermolysin	G/L	GL	804/805	Cleaves in MMP enzyme cleavage domain	Protein sequence	[110]
Thrombin	AGPR/GAA AGPR/GSP	AGPR/GAA AGPR/GSP	365/366 641/642	Cleavage	Protein sequence	[44]
Factor Xa	DGR/NG	DGR/NG	118/119	Cleavage	Protein sequence	[44]
Binding sites for extracellular matrix molecules, cells and other ligands						
Extracellular molecules						
Collagen I, II	N-terminal site unresolved KGHR KGHR IAGIGGEXA	IAGIGGEKA GPAGMPGFPGMKGHR (C-propeptide)	99–119, 1052–1068	Form heterotypic fibrils in tissue-specific manner	CNBr fragments and synthetic peptides with Western blots	[77]
Keratan sulfate proteoglycan (KSPG)	GERGEQGPAGS on collagen I	GDKGDTGPPGP	474–484	Proposed to regulate spacing between collagen fibrils	Protein sequence	[48]
	GDRGDAGPKGA on collagen 1	GDKGEPGGPGA	588–598	Proposed to regulate spacing between collagen fibrils	Protein sequence	[48]
Dermatan sulfate proteoglycan (DSPG)	GDKGESGPSGP on collagen I	GDKGEGGAPGL	624–634	Proposed to regulate spacing between collagen fibrils	Protein sequence	[48]
	GDRGEPGGPGP on collagen I	GERGETGGPGP	645–655	Proposed to regulate spacing between collagen fibrils	Protein sequence	[48]
	GDRGETGPAGP on collagen I	GDRGENGSPGA	879–889	Proposed to regulate spacing between collagen fibrils	Protein sequence	[48]
	GDRGETGPAGP on collagen I	GDRGESGPAGP	909–919	Proposed to regulate spacing between collagen fibrils	Protein sequence	[48]
Heparin	KGHR	KGHR	110–113 953–956	Inhibits angiogenesis	Solid phase binding assay, endothelial tube formation assays	[111,112]
Pigment Epithelium-derived Factor (PEDF, SERPIN F1)	MKGHRGFDGRNG IKGHRGFOGNOG	MKGHRGFDGRNG IKGHRGFPGNPG	109–120 952–963	Complexes with collagen in cornea and vitreous	Solid phase binding assay	[52,113]
Decoron (decorin core protein)	KXDGRGE on collagen I; AKGDRGE on collagen I	GKGDRGE KSGDGRE	877–883 907–914	Inferred from sequence, position and site on A clade map	Rotary shadowing, X Ray crystallography	[49]
Fibronectin-binding protein	Binds about 122 nm from C-terminus of collagen I	Undefined binding sequence	about 800	Links fibronectin and collagen III. Binds near MMP cleavage site and near decorin with which it interacts	Rotary shadowing, X Ray crystallography	[57]
Secreted protein Acidic and Rich in Cysteine (SPARC, BM40, Osteogenin)	GVMGFO GAAGFP	GVMGFO GAAGFP	423–428 717–722	Secreted glycoprotein important in mineralization	CNBr fragments, synthetic peptides and rotary shadowing	[46]
Thrombospondin	Binds close to both N- and C-termini	Undefined binding sequence(s)	Unknown	Platelet aggregation and antiangiogenic, mediates cell-cell and cell-ECM binding	Rotary shadowing	[58]
Tenascin X	Unknown	Unknown	Unknown	Binds to collagen III and decorin	Solid phase binding assay	[114,115]
Cells						
Integrin α1β1, α2β1	GRPGER (GROGER)	GROGER	84–89	High affinity, expressed on platelets	Rotary shadowing and synthetic peptides, collagen III toolkit	[60]
Integrin α2β1	GLPGER (GLOGER)	GLOGER	135–140, 150–155	High affinity	Rotary shadowing and synthetic peptides, collagen III toolkit	[61]
Integrin α2β1	GAPGER (GAOGER)	GAOGER	525–530	Low affinity	Rotary shadowing and synthetic peptides, collagen III toolkit	[61]
Integrin α2β1	GMPGER (GMOGER)	GMOGER	573–578	High affinity	Rotary shadowing and synthetic peptides, collagen III toolkit	[61]
Integrin α2β1	GLSGER	GLSGER	834–839	High affinity	Rotary shadowing and synthetic peptides, collagen III toolkit	[61]
RGD	RGD	RGD	938–940	Integrin- binding site, not usually relevant in native collagen	Protein sequence	[64]
DDR2 (Discoidin domain receptor -2)	GVMGFO	GVMGFP	423–428	Cell surface collagen receptor, tyrosine kinase receptor that activates fibroblasts	Synthetic peptides	[116]
LAIR-1 (Leukocyte associated Ig-like receptor-1)	(GPO)10	GAPGLRGGAGPPGPEGGKGAAGPPGPP GEGGPOGVAGPOGGSGPAGPOGPQGVK GPAGPAGAPGPAGSRGAOGPQGPRGDK	537–563 681–707 915–941	Inhibits immune responses, found on mononuclear cells and thymocytes	Solid phase binding to collagen III and (GPO)_10_ peptide	[65]
OSCAR (Osteoclast Associated Receptor)	GPPGPAGFPGAP GGPGAAGFPGAR	GPPGPAGFPGAP GGPGAAGFPGAR	651–662 714–725	Tyrosine kinase-coupled receptor that costimulates osteoclastogenesis	Solid phase binding to synthetic peptides and type II toolkit	[67]
nCAM1 (Neural cell adhesion molecule1); ICAM1 (Intercellular adhesion molecule 1)	Motif unknown	Unknown	One binding site	Membrane glycoprotein but also part of the ECM	Solid phase binding assays of ligand to native proteins	[117]
MAG (Myelin- associated glycoprotein)	Motif unknown	Unknown	Two binding sites	Membrane glycoprotein, but also part of the ECM	Solid phase radio-ligand binding assay	[117,118]
Hemostasis						
Von Willebrand Factor (VWF)	RGQPGVMGF	RGQPGVMGF	422–430	Multimeric glycoprotein, adheres to collagen and platelet surface receptors at high shear rate flow	Synthetic triple helical peptides and solid phase binding assay	[71]
Platelet fibrinogen receptor (IIb/IIIa)	PXXXD	PAGKD	72–76	Controversial, biological significance unknown	Protein sequence	[76]
Glycoprotein VI	GAOGLRGGAGPOGPEGGKGAAGPOGPO	GAPGLRGGAGPPGPEGGKGAAGPPGPP	537–563	Platelet membrane protein mediating collagen-induced aggregation	Platelet and GPVI receptor binding to collagens and triple helical peptides in solid phase assays	[78]
Platelet-binding octapeptide, kindlin-3	KOGEOGPK	KOGEOGPK	502–509	Mediates platelet adhesion independent of GpVI and GpIa/IIa	Platelet binding to collagen peptides in solid phase binding assay	[79]
Platelet-derived growth factor (PDGF)	Motif not known	Unknown	C terminal part of collagen III	Binds to a tyrosine kinase receptor to stimulate cell growth	Solid phase binding assay	[119]
Proteins of infectious agents						
Langerin	ASQNITYHCKNS	ASQNITYHCKNS in pro-peptide	N-terminal to triple helix	C-type lectin	Mass spectrometry	[82]
YadA (Yersinia adhesion A)	GPO to (GPO)6	(GPP)3	24–32	Outer membrane protein important in adhesion	Solid phase binding to synthetic triple-helical peptides	[85]
AAEL010235 Aegyptin	RGQPGVMGF (high affinity) (GPO)10, GFOGER (lower affinity)	Several	419–427	Anticoagulant from saliva of blood sucking insect, Aedes aegypti; inhibits binding of vWF, glycoprotein VI and α2β1integrin	Solid phase binding assay	[72,73]
Calin	Motif unknown	Unknown	About 419–427	Anticoagulant in leech saliva (Hirudo medicinalis)	Inhibits VWF binding to collagen III	[74]
Leech antiplatelet agent (LAPP)	Bovine GPPGPRGGAGPPGPEGGK	GAPGLRGGAGPPGPEGGK	537–554	Anticoagulant in Leech saliva	Inhibits VWF binding to collagen III	[75]
MIP (Macrophage infectivity potentiator protein)	Motif unknown	Unknown	Unknown	Virulence factor found in Legionella	Solid phase binding assay	[86]
Miscellaneous ligands						
G protein-coupled receptor 56 (GPR56)	Motif unknown	Unknown	Unknown	Orphan G-protein coupled receptor, important in cortical development	Solution phase “pull down” binding assay	[120]
PR47 (47 Kd platelet receptor for collagen III)	Motif unknown	Unknown	Unknown	Membrane receptor for collagen III similar to collagen I receptor	Solid phase binding assay	[121,122,123]

The heparan sulfate binding motif is KGHRGF in collagen III and there are two sites at the N and C-termini in the same locations as for collagen I. The heparan sulfate proteoglycans act as co-receptors for growth factors and are pro-angiogenic [50].

Pigment epithelium-derived factor (PEDF), also known as Serpin F1, has a variety of functions including being anti-angiogenic and anti-tumorigenic. It inhibits VEGF expression but upregulates that of thrombospondin [51]. It binds to the same residues in collagen III as heparin and heparan sulfate [52].

Decoron and probably biglycan bind at two sites in bands d and e in collagens I and III [49, 53]. Decorin also binds to fibronectin, complement component C1q, epidermal growth factor receptor (EGFR) and transforming growth factor β (TGFβ). Decorin binding to collagen III inhibits fibrillogenesis, but is pro-angiogenic [49, 54].

#### Collagens

Collagen III binds covalently to collagens I and II through cross-links at their N- and C-termini to form heterotypic fibrils [77], that are thinner than those comprising collagen I alone [55]. Collagen III lacks 3OH-P residues [25] which may impair its ability to extend laterally and determines its peripheral location in the fibril [55,77]. Notably, the collagen III triple helix is fifteen residues longer than that of collagen I or II but it is not known how this feature may affect the assembly, structure or function of heterotypic fibrils. In cartilage, collagen III copolymerizes via a trivalent cross-link with collagen II [56] through the same sites [77]. Collagen V often occurs together with collagen III but there is no evidence currently for intermolecular crosslinking.

#### Fibronectin

There is no evidence for direct binding of collagen III to fibronectin. However a fibronectin-binding protein interacts with collagen III at about residue 800, after taking into account the propeptide [57], which is the same location that fibronectin binds in collagen I and II. This means that collagen III potentially binds to fibronectin and decorin on the same monomer, near the sites for MMP cleavage and heparan sulfate binding. The proximity to the fibrillogenesis domain suggests a regulatory role of fibronectin in collagen III chain assembly and degradation [21], and in particular, MMP cleavage potentially separates the fibronectin- and decorin- binding sites.

#### Thrombospondin 4

Thrombospondin is found in extracellular matrix, but also in platelets, and it contributes to platelet aggregation and inhibits angiogenesis. Thrombospondin binds to the N- and C-termini of collagen III [58,59].

#### Integrins

Collagen III binds to the α1β1 and α2β1 integrins, and potentially a variety of cells. Most P residues in the collagen III α1 chain are hydroxylated, which enhances integrin binding. The collagen III α1 chain has three integrin binding sites including GRPGER [60], GLPGEN, a high affinity site for α1β1 [61], and GMPGER, of rather lower affinity for α2β1 than GRPGERV [62]. Two lower affinity sites, GAPGER [60,63] and GLSGER have also been described. The GAPGER motif at residues 525–530 in the cell interaction domain [60], is at the same location as the collagen I high affinity site, GFPGER.

The RGD archetypal integrin- binding motif is thought to be less important in collagen where the triple helical conformation renders it inaccessible [64]. However the single RGD site in collagen III is in the same location as in collagen I, and may be exposed after enzymatic cleavage and thus become available for ligation by some integrin receptors [64].

#### LAIR 1 and 2

LAIR-1 is an immune regulatory receptor expressed on mononuclear cells [65]. LAIR-2 blocks the binding of LAIR-1 to collagen III [66]. Both LAIR-1 and -2 bind to (GPO)_3_, and there are at least two sites for interaction on collagen III.

#### OSCAR

This co-stimulates monocytes through the Fc receptor and activates osteoclast formation, resulting in bone resorption. OSCAR binds to GPOGPAGFOGAO with GPOGPXGFX as the minimum motif, where P can be substituted by A [67]. This sequence overlaps with a dermatan sulfate binding site. There is a second possible OSCAR binding site, GGPGAAGFPGAR.

#### Other cell-binding motifs

Collagen III also binds to ICAM1 on endothelial and immune cells, and NCAM1 on neurons, glia, skeletal muscle and natural killer cells but the binding motifs are unknown.

### Major structural domains

These include the major ligand-binding regions 1, 2 and 3 (MLBR1, 2 and 3), a cell-interaction domain, a Gap, an MMP/enzyme cleavage domain and the fibrillogenesis domain.

#### MLBR 1, 2 and 3

MLBR1 is found in approximately the same locations in collagen III and collagen I, and both sites share many binding motifs and ligands. Both MLBR1 have motifs for integrin-binding, intermolecular crosslinking, angiogenesis, and heparin-binding.

However, MLBR2 on collagen III includes the hemostasis- binding sites on Monomer 2, and MLBR3 includes the cell interaction domain and several neighboring functional domains on Monomer 3. This contrasts with collagen I where MLBR2 (with the greatest number of ligand binding sites) is located on Monomer 4 and MLBR3 on Monomer 5.

Notably, only collagen I has binding sites for the ligands involved in mineralization: cartilage oligomeric matrix protein (COMP or thrombospondin 5) and phosphophoryn. Where collagen III is increased in diseased bone or dentin, the tissues are looser, less-mineralized, and heal poorly after fractures [68].

#### Cell interaction domain

The cell interaction domain of collagen III is relatively exposed and allows access to cell surface integrins [21,22], e.g., α2β1 integrin ligation of GAPGER [61], as well as sites for platelets and LAIR-collagen ligation (8,9). Collagen I has an α1β1/α2β1 integrin binding site, GFPGER, at this location.

#### Gap

The gap zone of collagen III is near the MMP/enzyme cleavage domain, and access to binding sites and functional domains therein may be prevented or modulated to a great extent by collagen III’s C-terminus, whereas its removal could allow access.

#### MMP/enzyme cleavage domain

This domain falls near the overlap/gap zone border. Generally, enzyme access to the collagen III fibril is limited except at this location, where a paucity of imino acids is proposed to yield a looser fibril structure to facilitate enzyme access [69].

#### Fibrillogenesis domain

Sites involved in protein folding, fibrillogenesis and fibril stabilization in both collagen III and I include the C-proteinase cleavage motif, disulfide knot, and cross-link sites.

### Functional and disease-associated sites

The interactome had sites that reflected the normal biological functions of collagen III (hemostasis, angiogenesis, cell-binding) and that were associated with disease (infection, ageing and diabetes, and genetic variants).

#### Hemostasis regulatory domains

The Hemostasis domain 1 is located on M2 in band d, and binds von Willebrand factor (vWF), and salivary proteins from biting insects.

vWF mediates platelet adhesion in damaged vessels, and has been implicated in angiogenesis, and cancer spread [70]. It may normally be inaccessible because of proteoglycan binding, but trauma may expose the subendothelial collagen III binding sites. vWF binds to a highly conserved motif (RGQPGVMGF) in collagen III and I [71]. After circulating platelets attach to collagen III through the vWF binding site, they may then engage the glycoprotein VI and integrin α2β1 sites.

This domain also contains motifs for the mosquito salivary protein aegyptin [72, 73], and salivary products from ticks and leeches (aegyptin, calin and rLAPP) [74]. Some of these anticoagulants bind directly to collagen III, blocking vWF binding [74,75]. Aegyptin from the salivary gland of *Aedes aegypti* mosquito binds to RGQPGVMGF in collagen III.

There is a predicted motif (PKGND) similar to the platelet fibrinogen receptor binding site (Glycoprotein IIb/IIIa) (PXXXD) near the vWF binding site and a PAGKD motif near the integrin α2β1 motif [76].

The Hemostasis domain 2 is located at the cell interaction domain, with binding sites for platelet receptors, glycoprotein VI, Type III collagen binding protein (TIIICBP; kindlin) and integrin α2β1 [75]. Glycoprotein VI and kindlin-3 are involved in platelet glycoprotein IIb/IIIa activation.

Glycoprotein VI binding may be mediated by (GPO)_4_ at the N-terminus of collagen III where P hydroxylation is required for binding [77] but fewer repeats and interrupted sequences, such as GPOGPEGGKGAAGPOGPO, are also effective [78]. Both sites are recognized by LAIR1.

Collagen III binding protein (TIIICBP; kindlin-3) may recognize a linear platelet-binding octapeptide (KOGEOGPK), located between the vWF and integrin-binding motifs [79], but its biological relevance is controversial.

The integrin α2β1 (Gp Ia/IIa) binding site facilitates platelet binding and activation through other receptors, notably Glycoprotein VI.

#### Angiogenesis

Major binding sites involved in angiogenesis are the same for collagen III and collagen I, and include sites for heparin, α2β1 integrin, vWF, fibronectin and decorin [80, 81]. All these except for vWF are located at the C-terminus, and are absent from the terminal fragment after MMP cleavage.

#### Infection

Collagen III has binding sites for a number of mainly adhesive proteins produced by infectious organisms. These include langerin, a C-type lectin, which binds to ASQNITYHCKNS, a motif found in collagen III and I propeptides [82]. Collagen III is also susceptible to cleavage by *Clostridium* collagenase at the LGPA motif [83, 84]. *Yersinia* adhesion A (YadA) binds to (GPO)_3_ [85] and several other sites, especially those rich in imino acids, or hydrophobic or uncharged residues. Aegyptin, calin and rLAPP, from mosquitoes, ticks and leeches, all bind at different collagen III sites.

Collagen III also binds to macrophage infectivity potentiator protein (MIP) which is a *Legionella* virulence factor [86]. Its binding site in collagen III site is unknown, and the motif in collagen IV (CPSGWS) is not present in collagen III [87].

#### Ageing and diabetes

Collagens are long-lived proteins, and glycation in diabetes and ageing results in advanced glycation end-products, stiffness and interference with ligand binding [11]. The main glycation sites in collagen I localize to K residues within bands c and d [22,88], which are conserved in collagen III. Glycation at residue 431 potentially interferes with the binding of vWF, SPARC and DDRs.

#### Genetic variants

There were 396 different missense variants in the *COL3A1* variant database affecting residues in the collagen III D-period. There were also three nonsense variants and a two-base deletion all in the terminal exon. This corresponded to a total of 400 variants at 254 unique locations affecting the D-period monomers.

**Fig 5:**
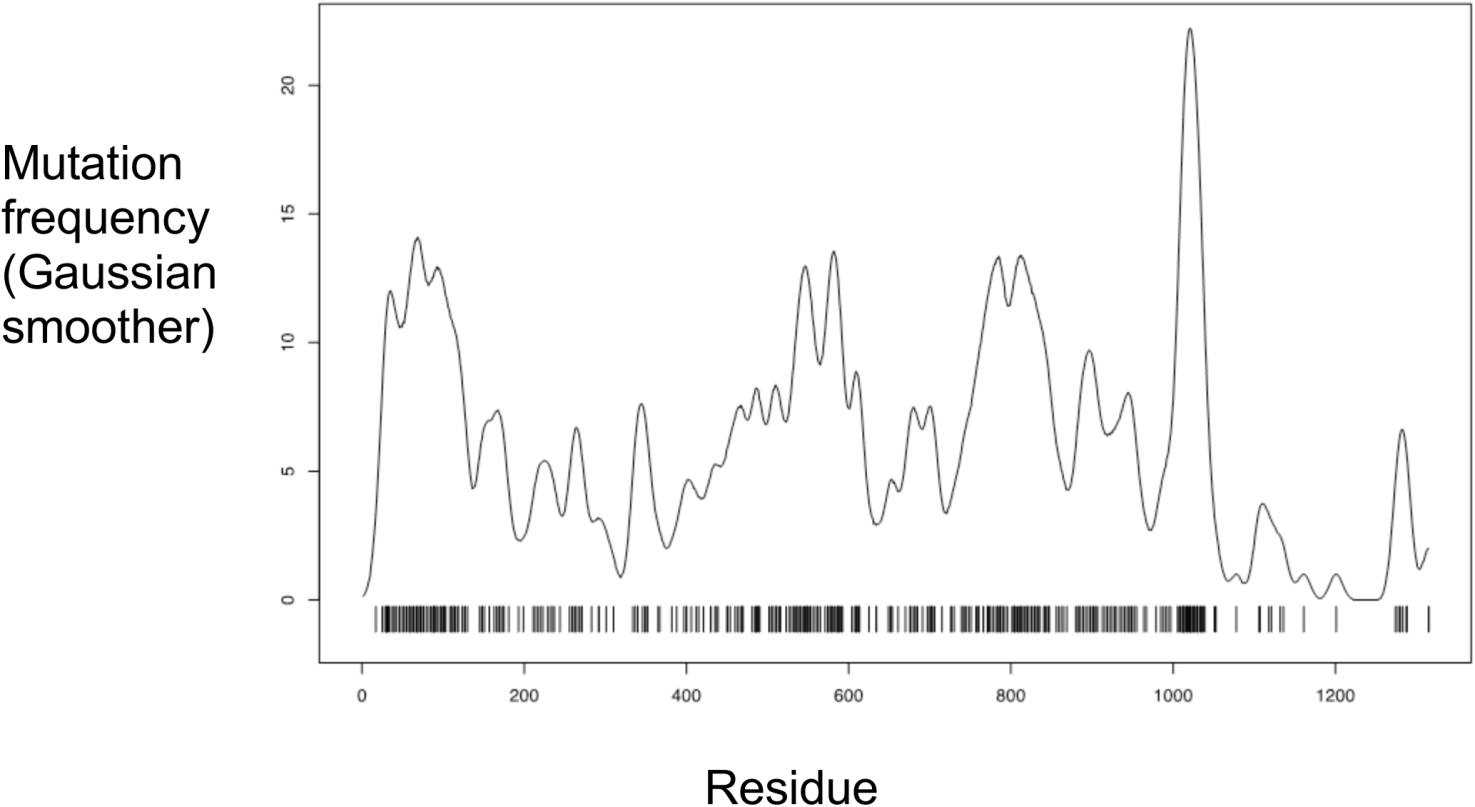
Missense mutation distribution in collagen III. Missense mutation frequency along the residues of the collagen III chain. This demonstrates the non-random distribution of variation.

Ten thousand random variant maps were generated each with 400 variants, randomly distributed throughout the collagen III α1 chain. The highest variant frequency peak was then calculated from each random map, and compared with the highest mutation frequency peak in the observed variant map (22.2). There were more frequent variants in residues 1000–1050 when repeated variants at the same residues were considered (Fig 5). This was also true for collagen I. None of the 1000 random maps had such a high peak variant frequency, and the observed variant peak was very significant, with a p value of < 0.0001. This increase was not significant when only unique locations were considered.

There were also fewer variants in the C-propeptide residues 1068 to 1315 compared with the randomly–generated variant maps (p<0.0001). None of the other 10,000 randomly-generated maps had a 250 residue window with 16 or fewer variants. There are fewer sequence variants in the C-propeptides than expected by chance (p<0.0001). This is also true for collagen I. The collagen III C-propeptide is critical in chain aggregation prior to triple-helix formation. Two chain-recognition sequences of 12 and 3 amino acids ensure that only procollagen III participates in triple helix formation. The sequences contribute to a complex three-dimensional structure comprising helices, β-strands and turns [30]. The C-propeptide includes a single N-linked glycosylation site, eight conserved C residues essential for the inter-chain disulfide bonds, and six residues for Ca^2+^ binding. Together, these represent 157 of the 245 C-propeptide residues. Counterintuitively, most of the few disease-causing variants in the C-propeptide do not affect residues in identifiable structural or functional motifs (S2 Table). However, amino acid substitutions, for example, at propeptide positions 92, 211 or 219, might disrupt the three-dimensional structure [89] but their pathogenicity is still unproven. Thus, substitutions at some residues in the C-propeptide may result in EDS, some in perinatal lethality, and others in a minimal or undetectable phenotype.

For both collagen III and I, the increased numbers of missense variants in the Cell interaction domain and C-terminus were congruent [21,22], but there was no obvious correlation between the apparently non-random distributions of variants and functionally important sites.

This study examined the effect of variant location, rather than variant type, on clinical phenotype. Already *COL3A1* variant type (G substitutions, splice site mutations, indels) are known to affect disease severity, being associated with an earlier age at onset of the first major complication [90].

Bruising is common with *COL3A1* missense variants, but massive hemorrhage is rare except with viscus rupture with vascular EDS, despite the two hemostasis domains found in collagen III. Collagen I also has a site for vWF, but binding is low affinity and bleeding does not occur with *COL1A1* variants.

Some *COL3A1* variants are associated with particular clinical phenotypes. *COL3A1* missense variants in residues 652–925 have been reported more often with acrogeria [91]. The p.A698T [18,20] near the glycoprotein VI binding motif and the cell interaction domain has been associated with bleeding cerebral aneurysms or pelvic organ prolapse [92]. Gastroesophageal reflux and hiatus hernia may both be linked to *COL3A1* [19] although no allele has yet been identified.

Other mutant genes that produce a vascular EDS-like phenotype often encode binding partners of collagen III. These include the collagen I α1 and α2 chains, collagen V α1 and α2 chains, tenascin X, lysyl hydroxylase and ADAMTS2.

## Discussion

The collagen III interactome demonstrates an almost identical arrangement of structural and functional domains as collagen I. Both D-periods have the same number and spacing of intermolecular crosslink sites, and the location of charged residues, and hence fibril banding, is largely congruent. In addition, many of the major structural and functional domains (cell interaction domains, fibrillogenesis and enzyme cleavage sites, and major ligand-binding regions) were found in the same locations. These similarities enable collagen III and I to assemble in register to produce heterofibrils and carry out common biologic functions. They also enable collagen III to be replaced by collagen I in embryogenesis and wound healing. Yet, a major difference between collagen I and III identified here relates to the potential for greater flexibility of collagen III, which makes it an ideal structural component of embryonic tissues, early wound healing, and distensible organs in the adult (vasculature, uterus, small bowel).

In some tissues the retention of the N-propeptide by collagen III is consistent with its inability to extend laterally, and its fibrils being smaller and more peripherally located in the heterotypic fibril than those of collagen I.

Clusters of disease-causing missense variants that may reflect residues necessary for molecular folding, fibril assembly, or ligand interactions, as well as variant-free regions map to the same locations in both collagens III and I [22]. Yet, the clinical phenotypes associated with variants in the collagen III and I genes are very different. Osteogenesis imperfecta is characterized by bone fragility, and vascular EDS by distensible organ rupture, acrogeria and bleeding. This suggests different tissue expression patterns and binding partners. Thus, for example, collagen I binds to proteins that are required for biomineralization, with the overlap zone of collagen I incorporating binding sites for COMP and phosphophoryn that are not present in collagen III.

The organ rupture seen in vascular EDS may result from increased collagen III in large arteries and hollow organs rather than altered elastic properties since collagen III does not bind to elastin [16]. The clinical features of acrogeria probably relate to reduced collagen III in subcutaneous tissues [93]. An association with uterine prolapse, hiatus hernia and gastroesophageal reflux, if substantiated, may reflect less abundant collagen III and increased tissue laxity [19,20]. Cumulative ‘wear- and- tear’ in an organ structurally dependent on collagen III may contribute to rupture in adult life.

Although both collagens III and I bind vWF, only *COL3A1* variants commonly produce bleeding. This is probably because collagen III has more hemostatic ligand binding sites and a higher affinity for vWF [71], and is more abundant in the vascular sub-endothelium and more accessible on the outer fibril surface. The collagen III α1 chain has several closely- related binding motifs for platelet proteins that are critical in platelet immobilization and located in the Cell interaction domain. These sites have not been demonstrated in type I collagen. In addition, the collagen III α1 hemostasis domain is about 100 residues from the vWF binding motif, and platelets bound to the Cell interaction domain may bind simultaneously to the large vWF multimer.

In conclusion, interactomes summarize our understanding of a molecule’s structure and function, suggest further interactions and roles, and help explain how missense variants produce clinical phenotypes. The collagen III interactome emphasizes its resemblance to collagen I, indicates how its architecture confers flexibility, and explains its role in hemostasis. These results may also have practical applications in the design of bioactive yet flexible extracellular matrix scaffolds for a variety of uses in medical devices.

## Experimental procedures

### Construction of the collagen III interactome

We constructed the interactome of a collagen type III D-period, examined its structural features, charge distribution, ligand-binding sites, and missense sequence variant distribution, and compared these features with those for collagen I.

The reference sequences for the human pro-collagen α 1(III) (UniProt P02461) and collagen α1(I) and α2(I) chains (UniProt P02452 and P08123 respectively) were used.

The collagen α1(III) chain is 1466 amino acids, with a signal peptide (23 aa), N-terminal propeptide (130 aa), collagenous sequence (1068 aa), and C-terminal propeptide (245 aa). Here we renumbered the amino acid from one D-period as 1 to 1068, with N- and C-telopeptides (14 and 25 aa respectively) and a triple helix (1029 aa) [21] corresponding to amino acids 154 to 1221 in the reference sequence. Numbers from the interactome [Reference sequence number -153], were used to describe variants so that numbering began at the start of the mature protein.

### Alignment of charge residues in collagen III and collagen I interactomes

The cross-link K residues in the collagen α1(III) chain were aligned with those in the collagen α1(I) and α2(I) chains, and the D-periods examined for positively- (R, K) and negatively- charged (E, D) residues.

### Analysis of fibril stability and structure

The distribution of atypical amino acid triplets, that confer flexibility such as GAA or GGY, and of GPP-rich regions that promote rigidity, was examined for randomness. The relationship of atypical and GPP triplets was then examined in the overlapping D-period regions. The numbers of atypical and GPP triplets were counted in ten ‘bins’ of equal size, starting from the N-terminus, summed over the D-period and compared.

The location of regions of high and low-stability within the collagen III D-period were examined using the Collagen Stability Calculator http://compbio.cs.princeton.edu/csc/[94].

### Sites related to structure, assembly, turnover, modification, cleavage and ligand-binding

Binding sites and structural motifs described for collagen III were identified using the search terms ‘collagen III’, ‘ligand’, ‘binding partner’ etc, from the scientific literature, open access web sites (UniProt, UCSC, Biogrid, Reactome, STRING, MINT, IntAct, and Ex-PASy Peptide Cutter, see later for websites), and the collagen I interactomes (,) [21,22].

In general, previously-reported ligand-binding sites were derived from experiments using the native collagen III molecule, triple helical mimetic peptides or fragments thereof, or from measurements based on rotary shadowing electron microscopy of type III collagen-ligand complexes assuming that type III collagen residues were spaced an average of 0.286 nm apart [95]. Binding sites on fragments and peptides derived from type III collagen were included because, *in vivo*, some ligands bind only to the denatured collagen.

The collagen III reference sequence was also examined for short motifs with biological functions described in other proteins (ELM, MnM databases).

### Functional and disease-associated sites

The map was examined for sites that reflected the normal biological functions of collagen III (hemostasis, angiogenesis, cell-binding, etc) and disease associations (infection, glycation, inherited disease).

Non-synonymous DNA missense variants were identified from a search of the open access *COL3A1* web databases (http://eds.gene.le.ac.uk/home.php?select_db=COL3A1) on 6 July 2016). The locations of missense variants was examined for randomness. A Gaussian kernel smoother was used to calculate a smoothed variant frequency at each residue in the coding sequence corresponding to the D-period [96]. Ten thousand random variant maps were generated using the number of unique variant locations in the protein sequence. The peaks found using the data from the *COL3A1* variant database distribution were compared with proportion of maps with a randomly-generated variant peak of this magnitude [97]. A similar randomization analysis was performed to examine the lack of variants in any region. These analyses were performed using the statistical software R [98].

## Supporting information

S1 Table Numbering systems for reference sequence and mature collagen III. This compares the systematic numbering and numbering from the start of the mature collagen as shown in the interactome.
(DOCX)

S2 Table C-propeptide features and seqeunce variants. This indicates features in the C-propeptide which is cleaved from the collagen III chain before it forms the interacome and the effect of pathogenic sequence variants.
(DOCX)
