# Supplementary material for: The collαgen III fibril has a “flexi-rod” structure of flexible sequences interspersed with rigid bioactive domains including two with hemostatic roles
Source: PLoS One. 2017 Jul 13;12(7):e0175582. doi: 10.1371/journal.pone.0175582 (PMC5509119; doi:10.1371/journal.pone.0175582)
Supplement: S1 Table — This compares the systematic numbering and numbering from the start of the mature collagen as shown in the interactome. (DOCX) [file pone.0175582.s001.docx]

# S1 Table. Numbering systems for reference sequence and mature collagen III*

| **Region** | **Length (residues)** | **Systematic numbering** | **Numbering from the start of the mature collagen as shown in the interactome** |
| --- | --- | --- | --- |
| Signal peptide | 23 | 1-23 |  |
| N-propeptide | 130 | 24-153 |  |
| N-telopeptide | 14 | 154-167 | 1-14 |
| Triple-helix | 1029 | 168-1196 | 15-1043 |
| C-telopeptide | 25 | 1197-1221 | 1044-1068 |
| C-propeptide | 245 | 1222-1466 | 1069-1313 |
| Total length: | 1466 |  | 1313 |

# *Data from UniProt P02461
