# Supplementary material for: The collαgen III fibril has a “flexi-rod” structure of flexible sequences interspersed with rigid bioactive domains including two with hemostatic roles
Source: PLoS One. 2017 Jul 13;12(7):e0175582. doi: 10.1371/journal.pone.0175582 (PMC5509119; doi:10.1371/journal.pone.0175582)
Supplement: S2 Table — This indicates features in the C-propeptide which is cleaved from the collagen III chain before it forms the interacome and the effect of pathogenic sequence variants. (DOCX) [file pone.0175582.s002.docx]

**S 2 Table.C-propeptide features and sequence variants***

| **Feature** | **Length** | **C-propeptide location** | **Systematic location (add 1221)** | **Mature protein location (add 1068)** | **Comment** |
| --- | --- | --- | --- | --- | --- |
| p.N1230K |  | 9 | 1230 | 1077 | VUS; No conservation at this position: *COL1A2* encodes K at this site |
| helix (TSLKSVNGQIESLI) | 14 | 15-28 | 1236-1249 | 1083-1096 |  |
| beta strand (GSRKN) | 5 | 32-36 | 1253-1257 | 1100-1104 |  |
| p.P1258S |  | 37 | 1258 | 1105 | Vascular EDS: P is conserved at this position |
| p.A1259T |  | 38 | 1259 | 1106 | Vascular EDS: A is conserved at this position |
| p.A1259V |  | 38 | 1259 | 1106 | Vascular EDS: A is conserved at this position |
| Cysteine 1 | 1 | 41 | 1262 | 1109 |  |
| helix (CRDLKFC) | 7 | 41-47 | 1262-1268 | 1109-1115 |  |
| Ca^2+^ coordination | 1 | 43 | 1264 | 1111 |  |
| Cysteine 2 | 1 | 47 | 1268 | 1115 |  |
| p.P1270T |  | 49 | 1270 | 1117 | Vascular EDS: *COL1A1* gene encodes S at this position |
| p.K1273R |  | 52 | 1273 | 1120 | Vascular EDS: *COL1A2* encodes S at this position |
| beta strand (SGEYWV) | 6 | 53-58 | 1274-1279 | 1121-1126 |  |
| Ca^2+^ binding | 1 | 59 | 1280 | 1127 |  |
| Ca^2+^ binding | 2 | 61-62 | 1282-1283 | 1129-1130 |  |
| p.G1284E |  | 63 | 1284 | 1131 | Vascular EDS: G is conserved at this position and substitution probably interferes with formation of adjacent disulfide bond and Ca^2+^ binding |
| Ca^2+^ binding | 1 | 64 | 1285 | 1132 |  |
| Cysteine 3 | 1 | 64 | 1285 | 1132 |  |
| helix (KLD) | 3 | 65-67 | 1286-1288 | 1133-1135 |  |
| Ca^2+^ binding | 1 | 67 | 1288 | 1135 |  |
| p.D1288V |  | 67 | 1288 | 1135 | Vascular EDS: D is conserved at this position and is involved in Ca^2+^ binding |
| beta strand (IKVFCN) | 6 | 69-74 | 1290-1295 | 1137-1142 |  |
| Cysteine 4 | 1 | 73 | 1294 | 1141 |  |
| turn (METG) | 4 | 75-78 | 1296-1299 | 1143-1146 |  |
| beta strand (ETCI) | 4 | 79-82 | 1300-1303 | 1147-1150 |  |
| Cysteine 5 | 1 | 81 | 1302 | 1124 |  |
| beta strand (NPL) | 3 | 84-86 | 1305-1307 | 1152-1154 |  |
| beta strand (NVPRK) | 5 | 88-92 | 1309-1313 | 1156-1160 |  |
| p.K1313R (Patient 4) ([Stembridge, et al., 2015](#_ENREF_100)) |  | 92 | 1313 | 1160 | Vascular EDS/ EDS III: K is conserved at this position and substitution might interfere with the beta strand at 88_92 |
| beta strand (SAE) | 3 | 99-101 | 1320-1322 | 1167-1169 |  |
| helix (FGES) | 4 | 107-110 | 1328-1331 | 1175-1178 |  |
| beta strand (SYG) | 3 | 118-120 | 1339-1341 | 1186-1188 |  |
| chain recognition sequence-1 (GNPELPEDVLD) | 12 | 120-131 | 1341-1352 | 1188-1199 |  |
| beta strand (PEL) | 3 | 122-124 | 1343-1345 | 1190-1192 |  |
| helix (EDVLDVQLAFLRLL) | 14 | 126-139 | 1347-1360 | 1194-1207 |  |
| p.Q1353H |  | 132 | 1353 | 1200 | No known phenotype even though Q is conserved. The variant allele is too common to be associated with EDS |
| chain recognition sequence-2 (SSR) | 3 | 140-142 | 1361-1363 | 1208-1210 |  |
| beta strand (ASQNITYHCKN) | 11 | 143-153 | 1364-1374 | 1211-1221 |  |
| N-linked glycosylation site | 1 | 146 | 1367 | 1214 |  |
| Cysteine 6 | 1 | 151 | 1372 | 1219 |  |
| turn (QASG) | 4 | 160-163 | 1381-1384 | 1228-1231 |  |
| beta strand (KLM) | 3 | 170-172 | 1391-1393 | 1238-1240 |  |
| beta strand (SNE) | 3 | 174-176 | 1395-1397 | 1242-1244 |  |
| beta strand (EFKAEG) | 6 | 178-183 | 1399-1404 | 1246-1251 |  |
| helix (SKF) | 3 | 185-187 | 1406-1408 | 1253-1255 |  |
| beta strand  (TVLED) | 5 | 190_194 | 1411_1415 | 1258_1262 |  |
| Cysteine 7 | 1 | 196 | 1417 | 1264 |  |
| beta strand (GEWSKTVFEYRTR) | 13 | 201_213 | 1422_1434 | 1269_1281 |  |
| p.K1426E |  | 205 | 1426 | 1273 | No known phenotype even though K is conserved at this position within beta strand 9 |
| p.F1429* |  | 208 | 1429 | 1276 | Vascular EDS: Results from loss of final 37 amino acids |
| p.R1432* |  | 211 | 1432 | 1279 | Vascular EDS: Results from loss of final 34 amino acids |
| p.R1432L (Patient 2) ([Stembridge, et al., 2015](#_ENREF_100)) |  | 211 | 1432 | 1279 | Classical or hypermobile EDS: R is not conserved at this position in different species nor in human collagen I, II, and III. |
| Helix (AVR) | 3 | 215_217 | 1436_1438 | 1283_1285 |  |
| p.P1440S |  | 219 | 1440 | 1287 | Vascular EDS:P is conserved at this position |
| p.P1440L (Patient 1) ([Stembridge, et al., 2015](#_ENREF_100)) |  | 219 | 1440 | 1287 | Vascular EDS:P is conserved at this position |
| beta strand (DIA) | 3 | 222_224 | 1443_1445 | 1290_1292 |  |
| beta strand (EFGVDVGPVCF) | 11 | 234_244 | 1455_1465 | 1302_1312 |  |
| cysteine 8 | 1 | 243 | 1464 | 1311 |  |
| p.*1467Qext*45 |  |  | 1467 |  | Vascular EDS: stop codon mutated resulting in 45 amino acid extension |
| p.*1467Lext*45 (Patient 3) ([Stembridge, et al., 2015](#_ENREF_100)) |  |  | 1467 |  | Vascular EDS: stop codon mutated resulting in 45 amino acid extension |

# *Data from UniProt P02461
